# Analysis of spatial dynamic of epizootic process of bluetongue and its risk factors

**DOI:** 10.14202/vetworld.2017.1173-1183

**Published:** 2017-10-04

**Authors:** Fayssal Bouchemla, Olga Mikhailovna Popova, Valerey Alexandrovich Agoltsov

**Affiliations:** 1 Department of Animal Disease, Veterinarian and Sanitarian Expertise, Faculty of Veterinary Medicine, Saratov State Agrarian University (N.I. Vavilov), Saratov, Russia; 2 Department of Food Technology, Faculty of Veterinary Medicine, Saratov State Agrarian University (N.I. Vavilov), Saratov, Russia

**Keywords:** bluetongue, extrapolation, outbreaks, regression model, risk factors

## Abstract

**Aim:**

The study was undertaken to find out the spatial dynamic occurrence and patterns of the global spread of bluetongue (BT) disease for the period from 1996 to 2016, as well as the assessment of the risk of occurrence and its spread in 2017-2018.

**Materials and Methods:**

Outbreaks (serum samples were collected from clinically healthy as well as suspected animals in infected points) were confirmed and reported officially by veterinary departments which represent different geographical regions in the world to World Organization for Animal Health. These reports explained that ELISA and polymerase chain reaction were used to identify the BT disease, taking in the account number of infected, dead animals, and focus of BT infection in all susceptible animals from 1996 to 2016. Once conventional statistical population was defined (an observational study), we had classified data as well as possible to answer to our aim, using descriptive statistics methods, including the test of the relationship between different epizootiological indicators.

**Results:**

The spatial dynamic study of BT’s occurrence and its spread in the world over the two past decades was presented by different epizootic indicators. The given analysis includes assessment and measurement of risk factors. It was built too, regression models, and allowed to put different forecasts on the different epizootic indicators in the years 2017-2018 by the extrapolation method. We had also determined that, in 2017, BT continues to spread with the total expectancy of 3.4 focus of infection (number of diseased animals in a single unfavorable point) and mortality of about 26 %; these rates tend to decrease in 2018. At abused points by BT, up to 78.4% of animals are mixed (more than one type) and in 21.6% - uniform. By this way, the relative risk of the incidence of appearance-abused points in mixed households has 3.64, which might be considered higher for the BT dissemination. Moreover, between the enzootic index and other epizootiological indicators had revealed an inverse correlation, i.e., to an increase in the level of enzootic index among the cattle population would be formed population less sensitive to BT. Cluster analysis was done, which had demonstrated the zoning of risk levels in the world and the occurrence of the disease intensity in the period 1996-2016 years. Then, assess connection degree of the dynamic of BT tension with geographical and socioeconomic conditions background 0.66 and 0.68, respectively.

**Conclusion:**

It is important to define a variety of BT risk factors and assess their influence on BT occurrence. However, the most important is to define the overlapping coinfluence between them that cause serious losses. To have an out of BT territory needs to make an emphasis of co-influence of risk factors on this zone. Was predicted a continue hits of disease in the next year with weight moderation through one year. Far from statists, to assess the given forecast may have a serious variety, taken in account problems of actual climate change in the world.

## Introduction

Bluetongue (BT) recorded by the World Organization for Animal Health (OIE), included in the list A, which relates to the number of highly dangerous and widespread infectious diseases in the world, has also significant socioeconomic issues in international trade [1-5]. According to the OIE data, BT dispersion is relevant in all continents, particularly, where developed sheep breeding. By the occurrence of BT, in the advantaged areas, morbidity and mortality rates sometimes reach 100%, with the possibility of stationarity of outbreaks [6,7].

A mass BT outbreak annually is estimated in minimum at 3 billion of dollars [8]. In India in 2005, the disease has covered 280,000 heads of sheep, in Italy (2013) more than 326,000 heads of sheep, with a mortality rate 40%, and in the neighboring country - Greece, in 2014, happened a similar situation [1,7,9-11].

It more than one and half century from scientific acquaintance with the first serotype of bluetongue virus (BTV), and until, this moment has been discovered 27 serotypes [12] and probably the 28^th^ serotype in South Africa recently (personal communication with Zientara Stéphan). By the time, BT gets spread in the entire world in a different incidence. In the last BT season had been reported more than 4700 outbreaks in 15 countries in the world (just in Europe 4560 outbreaks in 9 countries). BTV hurts more sheep and goats (in 92.9%) than cattle, although more than 64.28% of susceptible livestock in disadvantages points were cattle. In the past decades, dynamic of occurrence not only BT but also other transmissive diseases has shown an interesting evolution by time and spatial. It is why we focus our epizootical approach to determine BT factor of risk and make a schema to this situation and expected forecasts.

## Materials and Methods

### Ethical approval

Ethical approval was not applicable for this study.

### Samples collections

Outbreaks (serum samples were collected from clinically healthy as well as suspected animals in infected points) were confirmed and reported officially by veterinary departments which supervise conformed geographical regions in each country in the world to OIE. These reports explained that ELISA and polymerase chain reaction were used to identify the BT disease, taking into account number of susceptible, infected, dead animals, and focus of BT infection from 1996 to 2016 [10,13].

### Statistical analysis

The data on the different indicators of BT and their growth dynamic were processed according to the guidelines on conducting epidemiological monitoring of exotic, very dangerous, and unknown diseases, taking into account the OIE Animal Health Code [14,15] with the level of significance set at 5% to determine differences in test, and they were analyzed with regression using Microsoft Excel 2013. Cluster analysis was built with Excel 2013 too.

### Tests

Once conventional statistical population was defined (an observational study), we had classified data as well as possible to answer to our aim, and it was used.

Coefficient association test to define how connects animals holding manage with BT occurrence. Moreover, by this way, we could know not only the relative risk (RR) of the incidence but also we could have assessment and measurement of risk factors and more commonly used: Linear correlation coefficient: Rang r_s_ and Bravais-Pearson r. Used coefficients similarly show the dependence of the BT stationarity on incidence, focus of infection, and lethality as in cattle, as small cattle.

To calculate the connection degree of the dynamic of BT tension with geographical and socioeconomic conditions was used methods of hypothesis testing, criterion *χ*^2^, and calculation of the influence force of various systems of factors (entropy of random diversity and total entropy of the complex).

Regression models were used to put different forecasts on the different epizootic indicators in the years 2017-2018 by the extrapolation method.

The cartographical spatial dynamic study (cluster analysis) was conducted with Microsoft Excel 2013 and QuickMAP program (geoinformation systems).

## Results and Discussion

During the period 1996 - (April) 2016, BT has been registered in 12 countries of the African continent (with 3987 outbreaks), 28 countries of Europe (156993 outbreaks), 9 countries in Asia (5684 outbreaks), and <0.001% of the remaining outbreaks spread in the rest of the world. European countries captured up 94% of all outbreaks, where it had been infected 1.5 million animals with more than 28% lethality (Table-1).

**Table-1:** The global spread of bluetongue in the world from 1996 to 2016.

Continent	Number of disadvantaged countries (%)	Number of outbreaks (%)	Number of diseased animals (%)	Number of dead animals (%)
Australia	1 (2)	4 (0.002)	10 (0.001)	3 (0.001)
America	5 (9)	9 (0.005)	108 (0.005)	39 (0.008)
Asia	9 (16)	5684 (3.410)	547997 (27.568)	114918 (22.183)
Africa	12 (22)	3987 (2.392)	37120 (1.867)	9781 (1.888)
Europe	28 (51)	156993 (94)	1402575 (71)	393302 (75.921)
All the world	55 (100)	166677 (100)	1987800 (100)	518043 (100)

Using the available data, to study, the spatial dynamic of BT’s evolution process was formed a complex of cartograms and diagrams to describe the situation.

Visual analysis of cartograms (Figure-1a) leads to conclude that the highest risk of registered BT is characteristic of the Mediterranean region and South Africa, as well as in Western Europe and the Indian subcontinent, where the registered incidence was higher than the world average (240 outbreaks). In the Middle East, Russia, and South America, the probability of the disease occurrence was less than the average bar in the world. For the Russian Federation, regions with the highest risk of BT are generally situated in the European part, and for Canada, regions closed to the boundary with the United States (we have not had data on the USA).

**Figure-1a:**
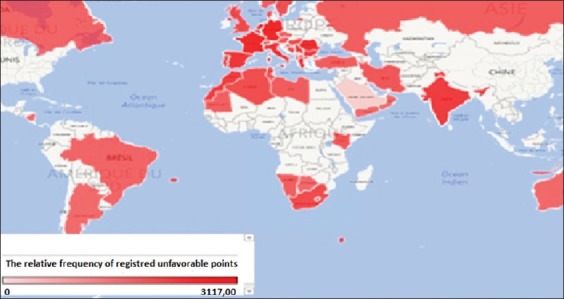
Clustering BT-risk areas in the world in the period from 1996 to 2016 years.

The highest risk of registering cases of BT (Figure-1b) is typical for the regions of Western Europe, Mediterranean, South Africa, and India, where the probability of above average registration of BT was more than 1783 heads. In America and Australia, the probability of occurrence of the disease had bellowed level face the average in the world.

**Figure-1b:**
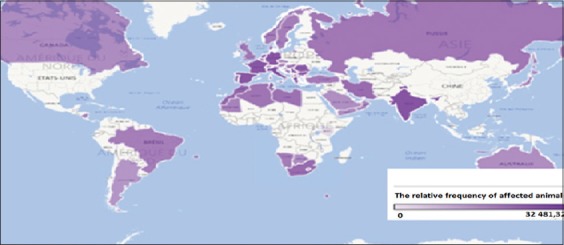
Cartogram BT cases in the world in the period from 1996 to 2016 years.

In Figure-1c, regions with the losses mind are India (more than 20% of word’s loss), the Mediterranean basin, and in recent years, Eastern Europe, where BT encompasses thousands of animals from season to another (average value 731.57 heads), as cattle as small ruminants were seriously infected. In the countries of northern Europe, Russia, and Middle East, losses reduced to 0.5 heads per season.

**Figure-1c:**
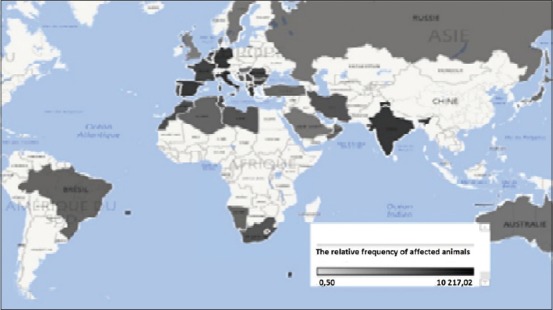
Clustering losses related to BT in the world in the period from 1996 to 2016 years.

Visual analysis of clustering with data about wild animals (Figure-1d) leads to the conclusion that the highest risk of BT registered among wild animals typical for Europe and South Africa in the first time, with a high risk of virus circulation among native wild populations, and in a less degree in India and Palestine, where the probability of virus isolation did not reach the world average value. In the central regions of America, Australia, and Africa, the risk of occurrences disease exists [1,4,10,16-19].

**Figure-1d:**
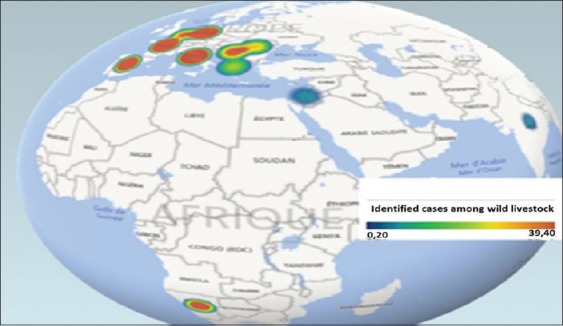
BT’s prevalence of the wild population in the period from 1996 to 2016 years.

The tension of the epizootic situation was identified too, by indicators of the incidence of cases and outbreaks in disadvantaged countries.

**Figure-2:**
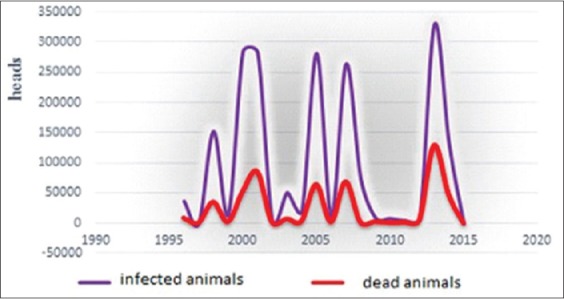
BT’s prevalence of the wild population in the period from 1996 to 2016 years.

Any variation of this distribution (Figure-2) is so important characteristics of the epizootic state of population. These variations can be a form of “dome” and “tails” (shallow and steep), uni- or polymodal (one or more packs), and asymmetry, i.e., the displacement of values on the chart in the increasing or decreasing direction expressing studied trait, limits, or volume of distribution, i.e., the range between the extreme values (as wider distribution as makes the more stable character).

From Figure-2, rather noticeably polymodal of the curve as wives, explaining the way to the emergence of the episodic BT process. It is also observed that the size of these waves has not a constant option (asymmetry), and their base closely varies in short intervals of 2-3 years. The nature assortment of the virus complicates, eradicates it, and gives another possibility of all sorts, including those in the new territories may explain this. In the same way, we cannot exclude the latent introduction.

The coincidence of the periods of decline and rise between defeat (infected heads) and lethality indicates a certain relation between themselves. It was confirmed with correlation coefficient (equal 0.94) - direct and strong link.

Periods of remission accompanied with a closed widespread; in 1997, BT was present only in 6 countries around the world, and in 2004-2005, in 8 countries, and vice versa, but conversely, in 2009-2011, more than 20 countries announced his disadvantaged by BT, and 15 countries in 2016.

Knowing the features of any disease is the key to its eradication and besides, if it is expected. Therefore, we tried to build regression models of different indicators to predict the expected future situation on 2017-2018 years.

Construction of curves for different indicators shows large variations that complicated our task in their approximation (for trend line) and may not allow for reliable forecasts.

One-off the test function is selected; the next step would be choosing the function parameters giving as possible a good approximation to the experimental points (approximation ratio R^2^). In the order to obtain, a high R^2^ value was counted up more moderate functions (smoothing averages over three consecutive points [or smooth down 5 if necessary]), which more appropriate in such case, to give a more moderate form of regression models that improve the value of R^2^.

Regardless of the R^2^ value, the use of the obtaining models was as an extrapolation to predict the expected situation. The results are shown in Tables-2 and 3 and Figures-3-5.

**Table-2:** Extrapolating results of epizootic outbreaks, infected animals in and death on 2017.

Prevalence	++
Outbreaks (points)	112747.69
Infected animals (heads)	379571.32
Dead animals (heads)	97597.51

**Table-3:** Predict of results by extrapolation models of morbidity, lethality, and focus of infection on 2017-2018 years.

Indicators	Animal species	2017	2018
Morbidity (%)	Small cattle	19.49	14.52
	Cattle	88.99	-
Lethality (%)	Small cattle	1.014	15.59
	Cattle	11.45	38.1
Focus of infection	Small cattle	-	-
	Cattle	148.2	-

-=Negative value

**Figure-3a:**
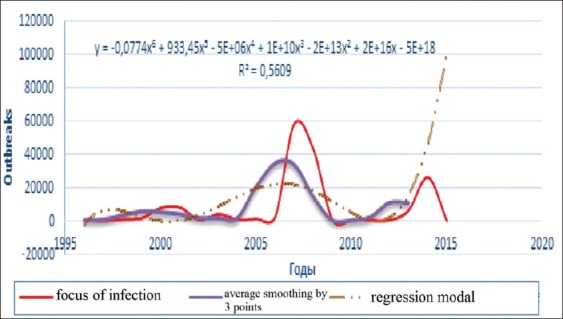
Dynamics of BT outbreaks in the period 1996-2016, average smoothing and its regression model.

**Figure-3b:**
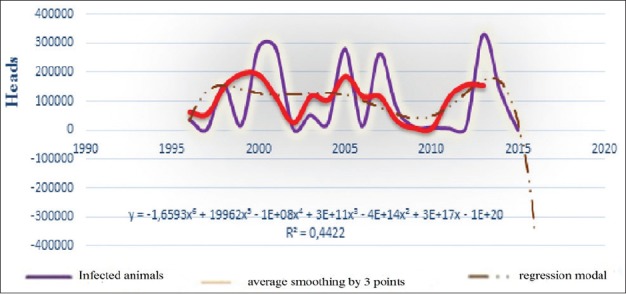
Evolutionary prevalence of BT, average smoothing and its regression model from 1996 to 2016 in the world.

**Figure-3c:**
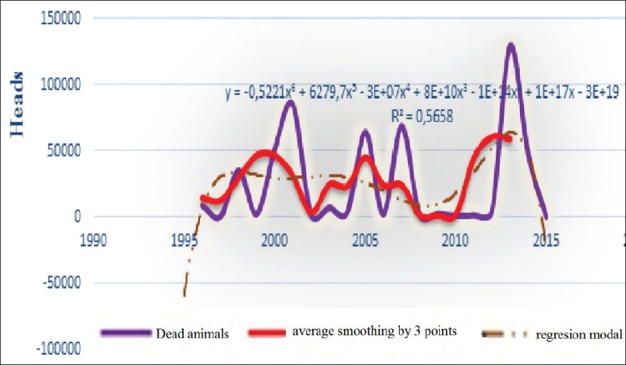
Dynamic of BT lethatity, average smoothing and its regression model from 1996 to 2016 in the world.

**Figure-4a:**
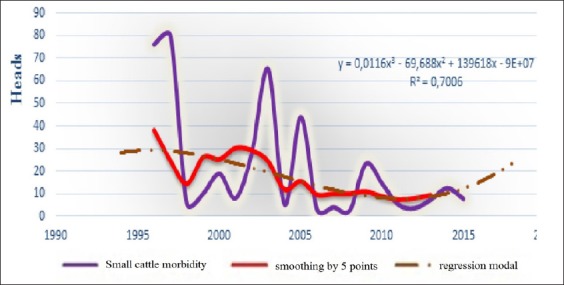
Dynamic, average smoothing and its regression model of the BT episodic morbidity in small cattle for the period 1996-2016 in the world.

**Figure-4b:**
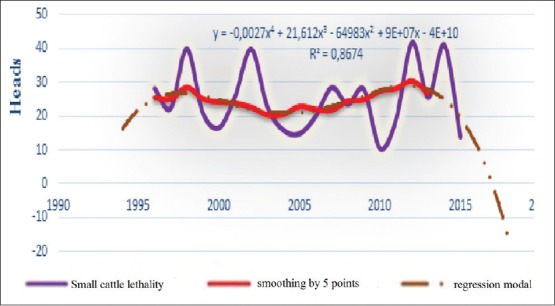
Chronology of dynamics, average smoothing and its regression model of the BT lethality in small cattle for the period 1996-2016 in the world.

**Figure-4c:**
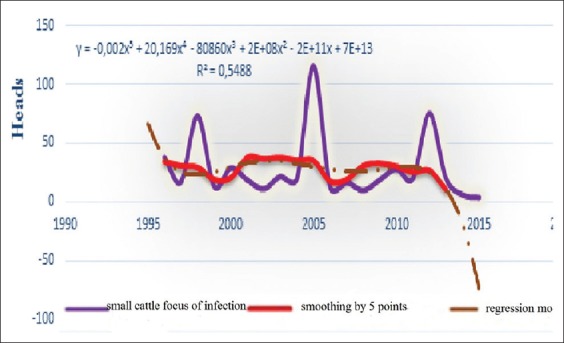
Dynamics of BT focus of infection, average smoothing and its regression model of the in small cattle for the period 1996-2016 in the world.

**Figure-5a:**
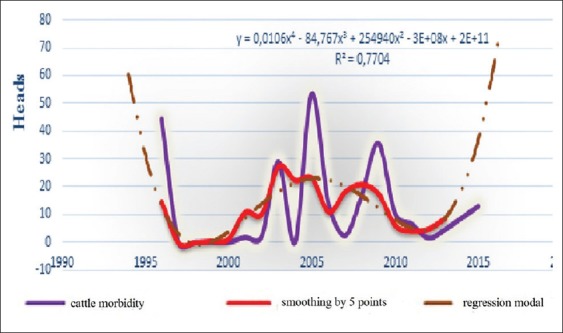
Dynamic, average smoothing and its regression model of the BT epizootic morbidity in cattle for the period 1996-2016 in the world.

**Figure-5b:**
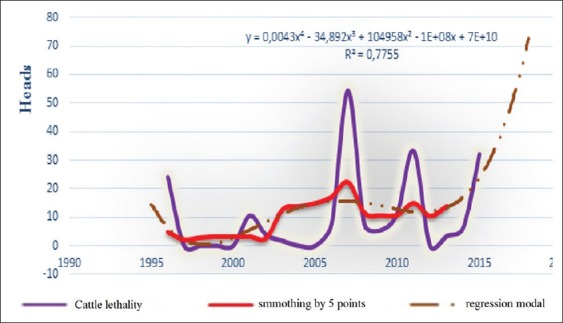
Chronology of dynamics, average smoothing and its regression model of the BT lethality in cattle for the period 1996-2016 in the world.

**Figure-5c:**
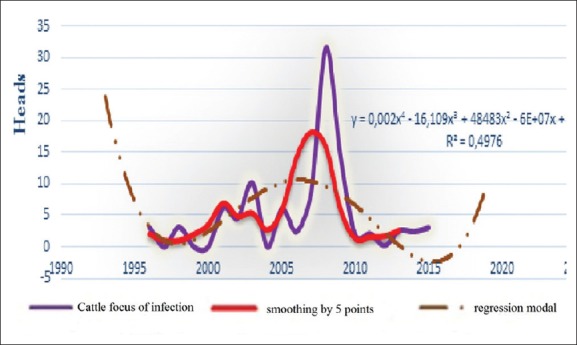
Dynamics of BT focus of infection, average smoothing and its regression model of the in cattle for the period 1996-2016 in the world.

All charts of the 3^rd^ figure demonstrate 4 large episodic: In 2000, 2005, 2007, and 2014, in which economic losses were very noticeable. Although chart’s progress varied with a less asymmetric multimodal that complicates build a regression model. Access to the average grading rules (smoothing) had helped building different models, whose extrapolation for 2017 posted in Table-2.

Seeing our previous analytics of the dynamics of BT prevalence and losses from until 2016, expected wave should be regenerate, and the results of this table have just been suit this dynamic. They can be compared with the packs of other waves. Hence, at the table, we may predict what will be expected from BT focus of infection with 3.37 and lethality rate around 25% in 2017. Moreover, all these indicators tend to decrease (not sharp) in 2018 globally.

Since BT affects small cattle, as well as cattle, and other species of wild population [4,5,10,13,14,19-28], we had tried to study separately the different expected epizootic parameters in small cattle (and cattle) in the following 2 years, to lodge with finest possible forecasts. The obtained results are shown in Table-3 and Figures-4 and 5.

From Figure 4a, may be concluded that the incidence index in small cattle was expressing freestyle different sizes (83-19), and with the base almost homogeneous (2-3 years). To avoid large variation of size was necessary building a medium-sized smoothing of 5 followed points (values):

Obtained model: Y_t_=0.0116χ^3^−69.688χ^2^+139618X−9E+07

Where Y_t_ *-* Value of models of the dynamics epizootic situation,

X - The serial number of the year.

R^2^=0.7 demonstrates a great approximation.

Model of theoretic values had comforted to 2014, where the value also increasing a bit and become 19.5% in 2016, and then, will reduce to 14.52% in 2018 (Table-3).

Interpretation of the curves of Figure-5a shows that curve asymmetric and not so polymodal. However, interested outbreaks can be registered forward. The cycle of corresponding model has varied from 12 to 15 years. It should be noted that this model has to rise in the near future.

In our case, it had seen that lethality had strong connection with mortality (correlation coefficient r=0.94), therefore, every change of one index, other index will automatically change. Especially, can see this link at comparison of these indices (for example, in the small cattle, Figure-4a and 4b). Furthermore, the same conclusions can be said about dynamics of conformed models, in 2017, expected “morbidity - lethality” in small cattle 19.5-1.49%, and in 2018, both reduced at 14.52% and 15.6% (Table-3).

The criterion of the focus of infection shows the intensity of infection in any disadvantaged point. Moreover, in this situation, these curves of both types of animals are different, which suggest that the emergence of BT process includes many factors, some of these have a strong influence (Table-4) and other - weight influence. Only, their interaction together can determine the type, the occurrence of episodic and even geographical distribution.

**Table-4:** The main suspects BT transmission established ways during the episodic outbreaks from 1996 to 2016 in the world.

Transmission way	Value	

	Absolute	%
Geographical proximity	46325	27.9
Trading of infected animals	147	0.09
BT infected wild animals	1879	1.13
Wintering (overwintering)	79869	48.1
Not installed (unknown)	38457	22.77
ε	166677	100

BT=Bluetongue

In addition, there is an internal interaction - between epizootic indices among themselves, with their reference to the weight influence factors (Table-5). They cannot generally specify the episodic cases about whose we discussed.

**Table-5:** The correlation coefficient (r) of epizootic indices interaction between themselves.

The ratio of the correlation index	r
Morbidity (small cattle) - Lethality (small cattle)	−0.060
Morbidity (cattle) - Lethality (cattle)	0.015
Morbidity (small cattle) - Focus of infection (small cattle)	0.146
Morbidity (cattle) - Focus of infection (cattle)	0.328
Lethality (small cattle) - Focus of infection (small cattle)	0.103
Lethality (cattle) - Focus of infection (cattle)	0.0787
Morbidity small cattle - Cattle	0.508
Lethality small cattle - Cattle	−0.117
Focus of infection small cattle - Cattle	−0.141

According to Table-3, it is well shown that the expected incidence of morbidity and focus of infection in both species in 2017-2018 will reduce but mortality rate, especially in small cattle, will be rising.

The main complexity of the BT epizootic resides in the length of the virus circulating in the reservoir of infection and the manifestation of the disease in all (epizootic, enzootic, and sporadic) forms, and their transitions one form to another.

Although BT sources and transmission paths are well known, in some cases, they could be so diverse, depending on the purpose and intensity of cultivation of animals, their description is only relevant to a concrete case (outbreak). Table-4 was created to determinate the participation share of BT factors in transmission.

Using available data from epizootic outbreak, investigations in different farms made an attempt to quantify the skidding BT ways. Information was gathered on the main routes of transmission of BTV, established at the epizootic outbreaks between 1996 and 2016 years (Table-4).

From Table-4, it is obvious that the degree of influence of various factors on the way of the distribution of BT in time is different seen that wintering is an important (48.1%) factor may qualify as an autosaved virus in cold periods. In our study, the majority of these outbreaks were registered in limited regions and time (2006-2008 years in the Europe), where the main pathogen (serotype 8) appeared with a new virulent approach (hit small ruminant, as well as cattle including wild animals).

Another important factor is considered too important in our opinion; this is the juxtaposition of BT infected points, which causes an increase in biological speed of transmission by vectors. It is the most speared factor in different countries in the world.

The role of trade and wildlife in epizootiology of BT is not significant (0.09% and 1.13%, respectively), for the first one due to the strict international legislative movement of live animals and their products, and for the second due to a two-fold reason: The difficulty of controlling and identifying incidents among them and their distribution.

It remains to be noted that 22.77% of cases had unknown cause for us, we had explained this by the no established (studied) factors of BT episodic process during the analyzed period (1996-2016 years). This fact indicates less level of system control in disadvantaged regions. Mainly, this is due to depopulation (active or passive), including wild animals, procurement and systems of rearing animals, especially in private farms (traditional).

Another factor, we tried to know how stationarity effect on BT tension (seeing enzootical factor - repeatability of BT occurrence (number of years) in a particular area to the total observation period). Table-6 presented the results of calculating the degree of stationarity influence on the tense epizootic situation.

**Table-6:** Correlation of stationarity effect - as a specific index of the incidence of cases and outbreaks of BT in cattle and small cattle in the world (1996-2016, statistical significance level a=0.05).

Indicators						

Stationarity	Morbidity		Lethality		Focus of infection	
		
	Small cattle	Cattle	Small cattle	Cattle	Small cattle	Cattle
[0-0.2]	7.61	17.92	33.26	19.62	9.25	5.01
[0.2-0.4]	11.45	3.25	29.71	12.09	19.55	6.87
[0.4-0.6]	9.83	12.91	36.23	3.11	13.19	174.66
[0.6-0.8]	14.31	4.37	29.37	0.12	35.81	4.7
[0.8-1]	7.66	3.51	22.02	6.96	48.44	4.25
The rang coefficient r_s_	0.3	−0.4	−0.7	−0.7	0.9	−0.6
Correlation coefficient r	0.17	−0.66	−0.68	−0.76	0.91	−0.01

BT=Bluetongue

The closeness of statistical relations may be determined by various factors (Fisher, Pearson, coefficient association, etc.), and more commonly used: Linear correlation coefficient: Rang r_s_ and Bravais-Pearson (r). Used coefficients similarly show the dependence of the BT stationarity on the studied indices.

The most significant direct correlation was found between the occurrence of the focus of infection among small cattle and the stationarity index rose r_s_=0.91; it is a very strong and direct connection. This dependence is due to the direct influence of enzootic factor on the occurrence of the focus of infection in small cattle and vice versa, reducing the recurrence of BT in the local population contributes to the reduction of livestock small cattle with a complex epizootic situation.

An average degree of dependence (r_s_=−0.6) takes place between the emergence of focus among the cattle and the stationarity index, i.e. to an increase in the level of the stationarity index among cattle formed population less susceptible to the disease (it serves as a vaccine).

A strong degree of indirect rang correlation r_s_=−0.7 (considered strong) was noted between mortality among cattle (small cattle too) and the stationarity index, which is due to the natural immunization of diseased animals during BT epizootic outbreaks was found too, a considerable correlation between the morbidity of cattle and stationarity r=−0.66.

It was revealed a weak positive correlation between the degree (r=0.3) of the dynamic of morbidity among small cattle and stationarity, due to the emergence of different serotypes of BTV. Although there is less cross-immune protection, apparently, it seems that there is an influence of other factors, no studied here.

Thus, it could be concluded that the stationarity index carries somewhere, character of natural vaccination, but with more severe losses.

## Effect of animal homogeneity breeding on the structure and the risk of BT

H_0_ null hypothesis testing procedure had been verified for data: (Chi-square test) *χ*^2^_obs_=54.83 and *χ*^2^_crit_=3.84. *χ*^2^_obs_ value is falling into the critical region: *χ*^2^>*χ*^2^_crit_, so the H_0_ hypothesis is rejected with an error probability of α 5%, and thus, both indicators are considered dependent. Mathematical calculations have confirmed that the connection to the BT susceptibility of small cattle is strong and direct (association rate=0.72).

Table-7 presented an analysis of the BT risk distribution of infected points by homogeneous breeding livestock (one or more kind of animals in holding) for the period from 1996 to 2016. In real terms, immediate risk (attributive or extensive) is the difference between the absolute indicators (morbidity) of control and experimental groups, and the RR - their ratio. It was found that the RR of BT in a small cattle equal 20.76 (immediate risk=11 and at odds=12.75).

**Table-7:** Analysis of the distribution of risks for BT infected points by homogeneous breeding livestock for the period 1996-2016.

Type of breeding	Outbreaks		Risk

	+	Total	
Mixed	130236	166034	R_1_=130236/166034=0.78
Homogeneous	35798	166034	R_0_=35798/166034=0.22

BT=Bluetongue

RRs frequency of BT infected points in the same period under the influence of factors - type of breeding (mixed or no) were greater than one and grew up to 3.64, which can be considered a high level of danger for spreading BT, with an excess risk (R_1_-R_0_=0.56) during the breeding of each species of animals separately.

## Analysis of the impact of socioeconomic and geographic conditions on the BT’s intensity

In carrying out, epidemiological analysis took into account a range of indicators of socioeconomic factors that reflect the level of socioeconomic development of regions [23].

The results of the analysis of the BT structural area derived from an assessment of the impact of geographical and socioeconomic factors background on the epizootic situation are given in Table-8.

**Table-8:** Impact of socioeconomic and natural conditions on the intensity of the BT epizootical situation (by the level of stationarity).

Factors	The power of influence
socioeconomic conditions	0.66
Geographic-natural conditions	0.68

BT=Bluetongue

Thus, it was established a statistically significant effect of these parameters on the stationarity index as direct and strong bond, suggesting conditionality. This dependence increases sharply with their synergy. According to the distribution of statistical calculations was found that the BT tensest situation has developed in the Mediterranean region.

## Conclusion

In the BT dynamics of small cattle, symmetry and polymodality are observed at all indexes, which indicate the homogeneity of the epizootic process, and conversely, in the case of cattle, there is an obvious heterogeneity. Participation of wintering phenomena with 48% in the BT occurrence does not make him the most important factor of risk, but the geographical proximity of infected holding may get it because of wintering which is limited in time and space.

According to the forecast, expected more and more of focus of infection with global moderate mortality up to 25% (nearly 97600 heads) in 2017 years, and these values tend to decrease (not sharp) in 2018 years. The marked correlation between all risk factors and its coinfluence defines degree in the really expected situation. The occurrence of BT disease on new territory may generate as new factors as a new serotype.

It should be emphasized here that such investigation study has to be checked in each geographical region to define local risk factors. Moreover, special attention should be taken to avoid or minimize vector spread reported by geographical proximity above as a main risk factor.

## Author’s Contributions

VAA and OMP designed the work. FB conducted the research work. Data analysis and manuscript were written by FB and OMP under the guidance of VAA. All authors read and approved the final manuscript.
